# Prognostic Value of Chest-Computed Tomography in Patients with COVID-19

**DOI:** 10.3390/arm90040041

**Published:** 2022-08-09

**Authors:** Gökhan Perincek, Canver Önal, Timor Omar

**Affiliations:** 1 Department of Chest Disease, Kars Harakani State Hospital, Kars 36200, Turkey; 2 Department of Radiology, Kars Harakani State Hospital, Kars 36200, Turkey; 3 Department of Cardiology, Faculty of Medicine, Kafkas University, Kars 36000, Turkey

**Keywords:** COVID-19, mortality, computed tomography, chest CT, prognosis

## Abstract

**Highlights:**

- Our study verifies that chest CT is one of the most helpful tools for diagnosing COVID-19 pneumonia.
- The chest CT scoring system shown in the current study provides important prognostic value in patients with COVID-19.
- The current study showed a correlation between chest CT score and inflammation biomarkers in COVID-19 pneumonia.

**Abstract:**

Background: The diagnostic value for chest CT has been widely established in patients with COVID-19. However, there is a lack of satisfactory data about the prognostic value of chest CTs. This study investigated the prognostic value of chest CTs in COVID-19 patients. Materials and Methods: A total of 521 symptomatic patients hospitalized with COVID-19 were included retrospectively. Clinical, laboratory, and chest CT characteristics were compared between survivors and non-survivors. Concerning chest CT, for each subject, a semi-quantitative CT severity scoring system was applied. Results: Most patients showed typical CT features based on the likelihood of COVID-19. The global CT score was significantly higher in non-survivors (median (IQR), 1 (0–6) vs. 10 (5–13), *p* < 0.001). A cut-off value of 5.5 for the global CT score predicted in-hospital mortality with 74% sensitivity and 73% specificity. Global CT score, age, C-reactive protein, and diabetes were independent predictors of in-hospital mortality. The global CT score was significantly correlated with the C-reactive protein, D-dimer, pro-brain natriuretic peptide, and procalcitonin levels. Conclusion: The global CT score could provide valuable prognostic data in symptomatic patients with COVID-19.

## 1. Introduction

Since 2020, the coronavirus disease2019 (COVID-19) pandemic, caused by severe acute respiratory syndrome coronavirus 2 (SARS-CoV-2), has been a significant global threat, with millions of victims [1]. COVID-19 occurs mainly in the form of an acute respiratory infection (pneumonia). The reverse-transcription polymerase chain reaction (RT-PCR) nucleic acid assay is the gold standard for clinical diagnosis [2]. However, the sensitivity of RT-PCR screening is relatively low under certain circumstances. The test may produce false negatives if the volume of the viral genome is insufficient or if the same temporal window of viral replication is missed [3].

Moreover, the RT-PCR process takes much time, and shortages of test kit supplies are common in some nations [4]. Chest-computed tomography (CT) might play a pivotal role in diagnosing and predicting the disease’s severity [5,6,7,8]. According to a growing number of reports, combined RT-PCR and CT imaging might assist healthcare professionals in diagnosing COVID-19 [8,9].

Countless reports have addressed the role of chest CT in COVID-19 disease. However, most of these studies included a smaller population or superficially rendered chest CTs [10,11,12]. Furthermore, there is a lack of satisfying data regarding the prognostic values of chest CTs in patients with COVID-19 [13]. Therefore, we investigated the prognostic values of chest CTs in hospitalized patients with symptomatic COVID-19.

## 2. Materials and Methods

### 2.1. Study Population

This single-center retrospective study reviewed the records of hospitalized patients with symptomatic COVID-19 from 12 April to 6 October 2020. We analyzed the demographics, comorbidities, laboratory results, and detailed chest CT findings. We included 521 adult patients who presented to our center within 96 h of the onset of symptoms. All patients tested positive for SARS-CoV-2 with confirmed RT-PCR. A 5-day favipiravir medication was administered for each subject. The study population was classified into two groups according to survival, and the findings were compared between survivors and non-survivors. Exclusion criteria were as follows: (1) Asymptomatic patients, (2) lack of chest CT, and (3) patients with erroneous or artifact chest CTs, which were not suitable for analysis.

### 2.2. Clinical and Laboratory Parameters

We collected data, including respiratory rate, heart rate, blood pressure, oxygen saturation, and laboratory findings at admission from hospital records. We analyzed the following biochemical and hematological parameters: albumin, total protein, C-reactive protein (CRP), D-dimer, ferritin, procalcitonin, and B-type natriuretic peptide (pro-BNP), white blood cell, lymphocyte, neutrophil, hemoglobin, platelet, and creatinine. Comorbidities of hypertension, diabetes, chronic obstructive pulmonary disease, coronary artery disease, cerebrovascular disease, chronic kidney disease, and chronic heart failure were interrogated. Smoking status was also checked. Chronic heart failure was defined as a left ventricle ejection fraction of <50%, and an estimated glomerular filtration rate (eGFR) of less than 60 mL/min was considered chronic kidney disease. The history of hemorrhagic or an ischemic cerebral attack was described as having a cerebrovascular disease.

### 2.3. CT Protocol

Chest CT images were obtained at the end-inspiration level with patients in supine positions with their arms elevated. Chest CT images of the patients were acquired using a 16-slice Toshiba Alexion CT scanner (Toshiba Medical Systems, Nasu, Japan). The scanning parameters were as follows: tube voltage = 120 kVP, automatic tube current modulation (30–80 mAs), pitch = 0.93–1.43 mm, matrix = 512 × 512 slice thickness = 2 mm, and field of view = 370 mm × 370 mm. All axial images with slice thicknesses of 2 mm were acquired with the fast scan method. All chest CTs were performed within 96 h of the onset of symptoms.

### 2.4. Chest CT Evaluation

A 7-year experienced radiologist (CÖ) retrospectively evaluated the CT images, blinded to the final diagnosis. Radiological terms, such as ground–glass opacity (GGO), crazy-paving pattern, and pulmonary consolidation, were based on the standard glossary for thoracic imaging reported by the Fleischner Society [14]. CT features were classified into three groups based on the likelihood of COVID-19 pneumonia according to the Radiological Society of North America Expert Consensus Statement [15] (1) typical, (2) indeterminate, and (3) atypical. The typical group included “bilateral, peripheral and basal GGO with or without consolidation”, “bilateral, peripheral and basal consolidation”, “multifocal and rounded GGO with or without consolidation”, “peribronchialenlargement”, “halosign”, “reversed halo sign”, “vascular enlargement” and “crazy-paving pattern”. The indeterminate group covered “unilateral GGO with or without consolidation”, “perihilar GGO with or without consolidation”, “diffuse GGO with or without consolidation”, “few and small non-peripheric GGO” and “perihilar consolidation”. The atypical group included “lobar pneumonia”, “bronchopneumonia”, “pleural effusion”, “interlobular septal thickening”, “pulmonary fibrosis” and “lymphadenopathy”.

**Figure 1:**
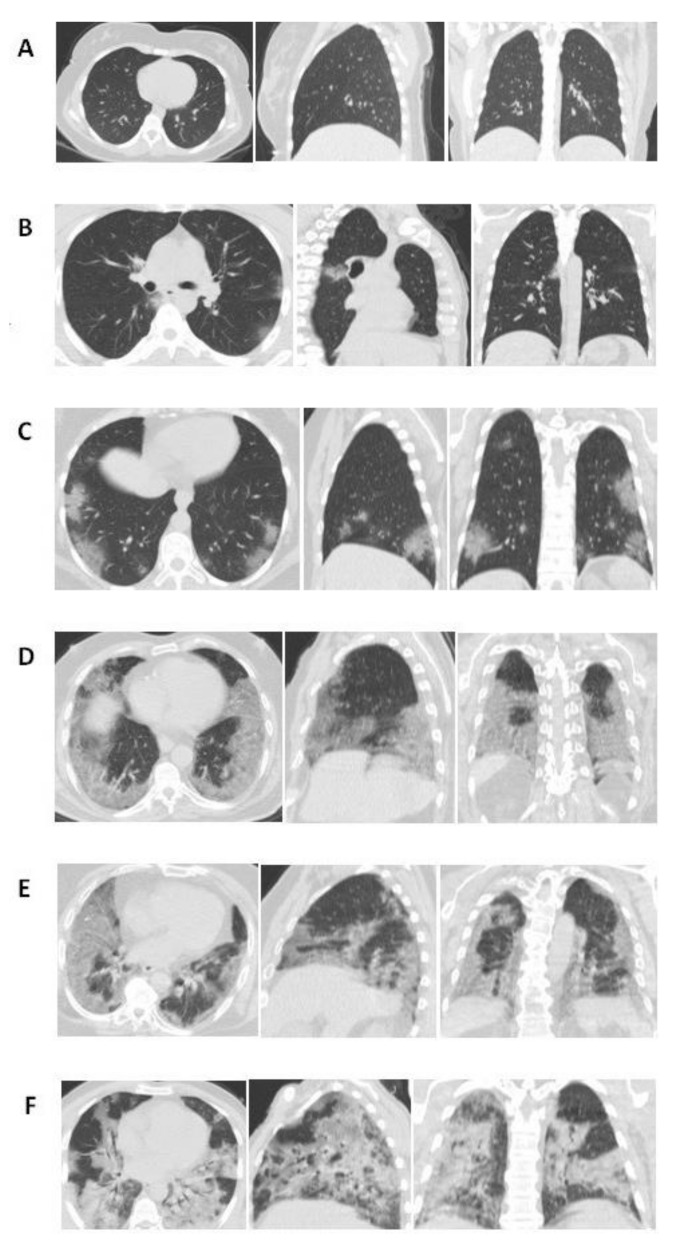
Chest CT findings of COVID-19 showthe CT score of right lower lobe involvement on axial, sagittal, and coronal images. (**A**) Score 0, 0% involvement; (**B**) Score 1, <5% involvement; (**C**) Score 2, 5–25% involvement; (**D**) Score 3, 26–49% involvement; (**E**) Score 4, 50–75% involvement; (**F**) Score 5, >75% involvement. CT: Computed tomography.

Furthermore, a semi-quantitative CT severity scoring system proposed by Pan et al. [11] was applied to each subject. By this system, each of the five lung lobes was assessed and given a score from 0 to 5, according to the extent of lobar involvement (0: no involvement, 1: involvement less than 5%, 2: 5–25% involvement, 3: 26–49% involvement, 4: 50–75% involvement, 5: involvement more than 75%). Each subject’s final global CT score was the sum of each lobar score, from 0 to 25 (Figure 1).

### 2.5. Statistical Analysis

Data obtained from this study were evaluated by using the SPSS 20 program. The normality test was maintained by using the Kolmogorov–Smirnov test. All continuous data in the study were not normally distributed, expressed as the median with the interquartile range. Categorical data were expressed as percentages and analyzed using the chi-square test. The Mann–Whitney U-test was used to analyze continuous variables. The statistical significance level was accepted as *p* < 0.05. A multivariate logistic regression analysis was performed to identify the independent predictors of in-hospital mortality, using variables showing significant associations in the univariate analysis. The receiver operating characteristic (ROC) curve analysis was also used to detect the optimum cut-off value of the global CT score for predicting in-hospital mortality. Additionally, a correlation between blood markers and the global CT score was assessed using Spearman’s correlation test.

#### Ethics Approval

The study protocol was approved by the Ethical Committee of Kafkas University and the health ministry (reference number 80576354-050-99/276; date of approval: 4 November 2020).

## 3. Results

### 3.1. Patient Characteristics

Of 521 patients (median age (IQR), 66 years (51–78)), 267 (51.2%) were male, 309 (59.3%) were discharged with recovery, and 212 (40.7%) expired throughout the hospitalization. The mortality rate was higher in males compared to females (63.7% vs. 36.3%, *p* < 0.001). Table 1 compares the demographic and clinical characteristics between survivors and non-survivors. Non-survivors were older than survivors [51 years (31–66) vs. 78 years (66–87.5), *p* < 0.001]. The most prevalent symptoms at presentation were dyspnea (55.5%), cough (45.5), fatigue (42%), arthralgia/myalgia (36.1%), and fever (27.1%). Fever, cough, dyspnea, fatigue, nausea, anosmia, anorexia, ageusia, sore throat, abdominal pain, headache, and arthralgia/myalgia were more common in non-survivors than survivors (all *p* values < 0.05). The most frequently recorded comorbidities were hypertension (46.1%), chronic obstructive pulmonary disease (COPD) (29%), and diabetes (16.9%). Frequencies of comorbidities (hypertension, diabetes, coronary artery disease, chronic heart failure, COPD, chronic kidney disease (CKD), cerebrovascular disease (CVD)), and smoking were higher in non-survivors (all *p* values < 0.05).

### 3.2. Results of Laboratory Characteristics

Considering laboratory findings, pro-BNP, CRP, procalcitonin, and D-dimer levels were significantly higher in non-survivors than survivors (median (IQR), 37.5 (13–137) vs. 555 (90–1936), 4.2 (2–10.5) vs. 79.7 (35.6–150.3), 0.052 (0.037–0.073) vs. 0.233 (0.117–0.589), and 343 (224–802) vs. 924 (324–157), all *p* values < 0.001, respectively). Ferritin was significantly lower in non-survivors than survivors (128 (42–254) vs. 439 (186–1053), *p* < 0.001). Despite being in normal ranges, WBC, creatinine, and neutrophil levels were higher in non-survivors than survivors. However, hemoglobin, lymphocyte, platelets (PLT), total protein, and albumin showed significantly lower medians(all *p* values < 0.001) (Table 1).

#### Results of Chest CT

**Figure 2:**
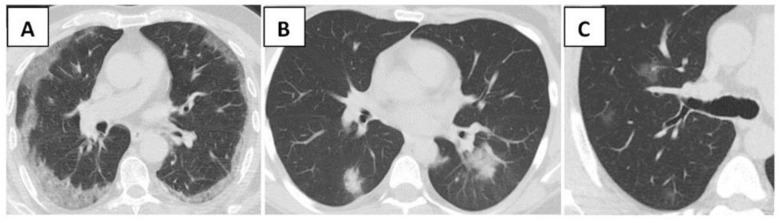
Chest CT findings of COVID-19. Transaxial images show (**A**) Bilateral, peripheral GGO; (**B**) Bilateral consolidation; (**C**) Multifocal rounded GGO. CT: computed tomography, GGO: ground-glass opacity.

According to chest CT, 411 (78.9%) patients had lung involvement. A total of 316 (60.7%) patients showed typical CT features based on the likelihood of COVID-19, while indeterminate and atypical features were found in 61 (11.7%) and 34 (6.5%) patients, respectively (Figure 2). The comparisons of the CT features between survivors and non-survivors are presented in Table 2. In the typical group, the most common disease pattern included bilateral, peripheral, and basal GGO with or without consolidation, and it was significantly higher in non-survivors than survivors (45% vs. 76.4%, *p* < 0.001). The second common pattern was bilateral, peripheral, and basal consolidation (11.1%), also significantly higher in non-survivors than survivors (8.7% vs. 14.6%, *p* = 0.036). However, multifocal rounded GGO with or without consolidation (4.8%) and the crazy paving pattern(1.9%) showed significantly lower proportions in non-survivors (*p* values, 0.01 and <0.001, respectively). Peribronchial enlargement and the reversed halo sign presented similar rates between the two groups (*p* values, 0.108 and 0.209, respectively). No halo sign and vascular enlargement were exhibited as CT findings. The proportion of patients who showed indeterminate features was similar between survivors and non-survivors (*p* = 0.058). In the atypical group, the most commonly found disorder, effusion (3.1%), was higher in non-survivors than survivors (0% vs. 7.5%, *p* < 0.001).

For features based on lesion localization and lobar involvement, the pathologies were as follows: LUL in 305 patients (58.5%), LLL in 299 patients (57.4%), RUL in 297 patients (57%), RLL in 250 patients (47.9%), and RML in 242 patients (46.4%). All were higher in non-survivors (all *p* values < 0.001). The global CT score median was 5 (IQR, 0–10), and it was significantly higher in non-survivors than survivors (median (IQR), 1 (0–6) vs. 10 (5–13), *p* < 0.001).

According to the univariate analysis, variables, including “age”, “gender”, “bilateral, peripheral and basal GGO with or without consolidation”, “bilateral, peripheral and basal consolidation”, “multifocal rounded GGO with or without consolidation”, “crazy paving pattern”, “typical CT involvement”, “unilateral GGO with or without consolidation”, “atypical CT involvement”, “effusion”, “total CT score”, “smoking”, “COPD”, “hypertension”, “coronary artery disease”, “chronic heart failure”, “diabetes”, “CVD”, “CRP”, “D-dimer”, and “pro-NBP” were significantly associated with in-hospital mortality. Thus, we put these variables in the multivariate analysis. The global CT score, age, CRP, and diabetes were independent predictors of in-hospital mortality (Table 3).

**Figure 3:**
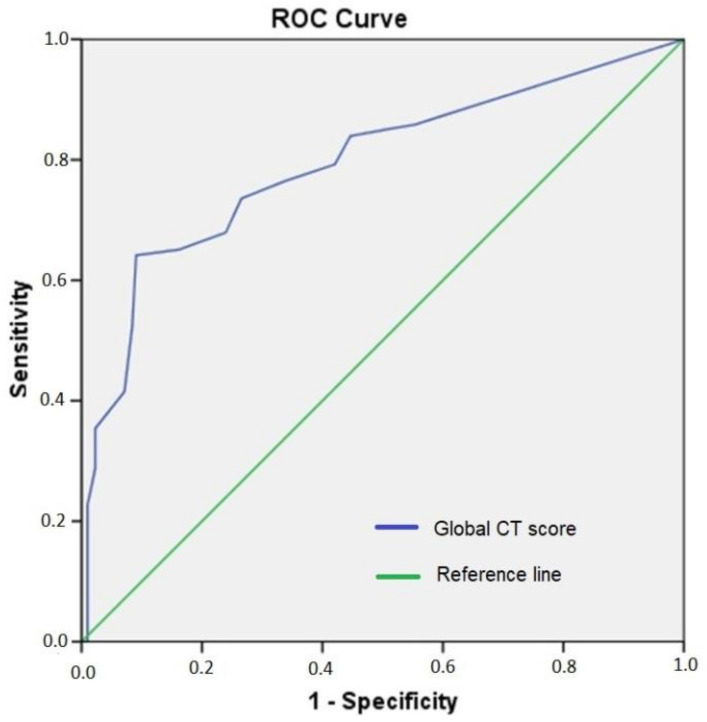
Diagnostic accuracy of the global CT score on in-hospital mortality in symptomatic patients with COVID-19 by the ROC curve.

**Figure 4:**
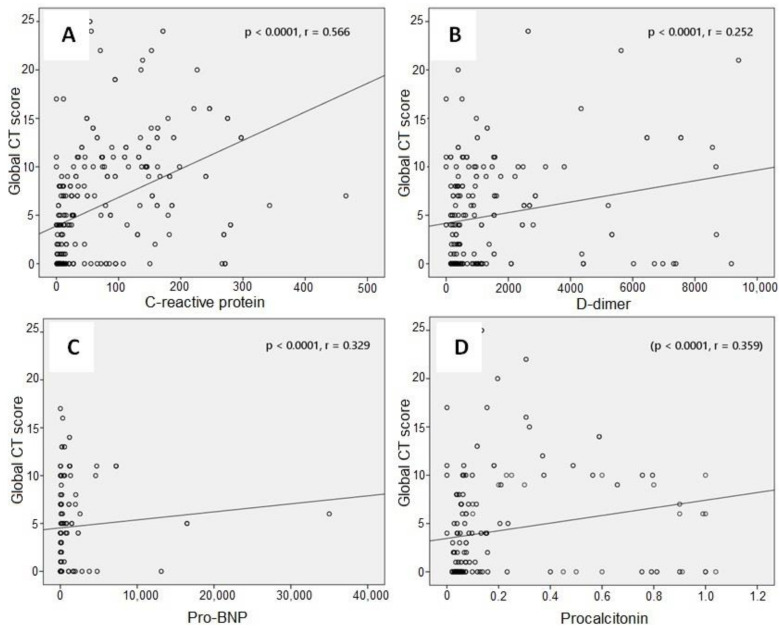
Correlation graphics between global CT score and C-reactive protein (**A**), global CT score and D-dimer (**B**), global CT score and pro-BNP (**C**), and global CT score and procalcitonin (**D**).

The optimal cut-off value of the global CT score for in-hospital mortality was 5.5 (with a sensitivity of 74% and specificity of 73% (area under the curve (AUC): 0.794; *p* < 0.001)) (Figure 3). Concerning correlation with blood markers, the global CT score was significantly associated with CRP (*p* < 0.0001, r = 0.566), D-dimer (*p* < 0.0001, r = 0.252), pro-BNP (*p* < 0.0001, r = 0.329), and procalcitonin (*p* < 0.0001, r = 0.359) levels (Figure 4).

## 4. Discussion

We investigated the prognostic values of chest CT features in hospitalized symptomatic COVID-19 patients. Our study showed that the global CT score could provide valuable predictive data for these patients. The global CT score, age, CRP, and diabetes were independent predictors of in-hospital mortality.

Consistent with prior reports [16,17,18], the most common CT findings in our study involved typical involvements, including bilateral, peripheral, and basal GGO with or without consolidation, bilateral, peripheral, and basal consolidation, multifocal rounded GGO with or without consolidation, peribronchial enlargement, and a crazy paving pattern.

Francone M et al. [19] studied a CT scoring methodology in patients with COVID-19. The CT score was significantly higher in critically ill patients than in mild disease patients. It was also significantly higher among late-phase than in early-phase patients. They found a significant correlation between CT score and CRP and D-Dimer. Similarly, our study showed that the median global CT score was significantly higher in non-survivors compared with survivors and that the global CT score was positively correlated with blood markers of CRP, D-dimer, pro-BNP, and procalcitonin.

Moreover, levels of these markers were increased in COVID-19 patients and strongly associated with outcome, as revealed in priorly published reports by Lippi G et al. [20], Lui F et al. [21], and Marin BG et al. [5]. In another study, Yuan et al. [22] investigated the characteristics of chest CT in COVID-19 patients. They also determined a CT score similar to ours. Non-survivors showed a higher CT score when compared to survivors. However, this study had a limited number of participants (only 27).

In the current study, pulmonary fibrosis and interlobular thickening were found in a few patients. Presumably, it was due to imaging times performed in the early stage of the disease. The longer the time between the onset of symptoms and the acquisition of the CT scan, the higher the probability of detecting fibrotic alterations indicative of late-stage pneumonia [23].

Our mortality data verified the prominent prognostic significance of age and the male gender [5]. Moreover, elevated heart rate and respiratory rate, low saturation and blood pressure levels, higher frequencies of coronary artery disease, chronic heart failure, chronic kidney disease, diabetes, COPD, and CVD were significantly correlated with mortality, as established by previous reports [2,24,25].

## 5. Limitations

Marked limitations of our study were:(1) We excluded asymptomatic patients, so we could not link our findings to all COVID-19 patients. (2) We lacked knowledge regarding discharged patients’ outcomes. (3) We only assessed chest CTs on admission; herewith, we could not provide data about the progression of the disease. (4) Since we lacked detailed data on pharmacological therapy for comorbidities, we could not calculate how treatment affected survival in-depth. (5) Although the radiologist was blinded to the final diagnosis, he was aware that the chest CTs he interpreted were suspects of COVID-19. Future prospective studies with large populations are expected to clarify the prognostic value of chest CT features in this field.

## 6. Conclusions

Our data show that as non-invasive and rapid imaging modalities, chest CTs might play a significant role in recognizing high-risk COVID-19 patients. Moreover, the global CT score could provide valuable prognostic data in symptomatic patients with COVID-19. Our study verifies prior reports concerning the diagnostic role of chest CTs in patients with COVID-19.

## Figures and Tables

**Table 1:** Demographic, clinical, and laboratory characteristics.

	Overall (*n* = 521)	Survivors (*n* = 309)	Non-Survivors (*n* = 212)	*p*-Value
Male, *n* (%)	267 (51.2)	132 (42.7)	135 (63.7)	<0.001
Female, *n* (%)	254 (48.8)	177 (57.3)	77 (36.3)	<0.001
Age (years), median (IQR)	66 (51–78)	51 (31–66)	78 (66–87.5)	<0.001
Initial Vital Signs				
SBP (mmHg), median (IQR)	110 (110–120)	120 (110–130)	110 (95–120)	<0.001
DBP (mmHg), median (IQR)	70 (60–70)	70 (70–80)	60 (60–70)	<0.001
Heart Rate, median (IQR)	96 (82–122)	82 (78–92)	126 (122–130)	<0.001
Saturation (%), median (IQR)	90 (80–94)	94 (93–96)	76 (72–80)	<0.001
RR/minute, median (IQR)	22 (20–30)	20 (18–20)	31 (28–32)	<0.001
Symptoms at arrival				
Fever, *n* (%)	141 (27.1)	54 (17.5)	87 (41)	<0.001
Cough, *n* (%)	237 (45.5)	109 (35.3)	128 (60.4)	<0.001
Dyspnea, *n* (%)	289 (55.5)	97 (31.4)	192 (90.6)	<0.001
Fatigue, *n* (%)	224 (43)	76 (24.6)	148 (69.8)	<0.001
Nausea, *n* (%)	26 (5)	24 (7.8)	2 (0.9)	<0.001
Diarrhea, *n* (%)	9 (1.7)	8 (2.6)	1 (0.5)	0.068
Anosmia, *n* (%)	15 (2.9)	13 (4.2)	2 (0.9)	0.029
Anorexia, *n* (%)	87 (16.8)	15 (4.9)	72 (34)	<0.001
Ageusia, *n* (%)	17 (3.3)	16 (5.2)	1 (0.5)	0.003
Sore throat, *n* (%)	59 (11.3)	52 (16.8)	7 (3.3)	<0.001
Abdominal pain, *n* (%)	10 (1.9)	9 (2.9)	1 (0.5)	0.046
Headache, *n* (%)	85 (16.3)	66 (21.4)	19 (9)	<0.001
arthralgia/Myalgia, *n* (%)	188 (36.1)	49 (15.9)	139 (65.6)	<0.001
Laboratory findings at admission				
Hgb (g/dL), median (IQR)	13.8 (12.4–15.3)	14.1 (12.8–15.7)	13.8 (10.2–14.7)	<0.001
WBC (×10^3^/μL), median (IQR)	6.9 (5.2–10.6)	5.7 (4.08–7.2)	7.35 (4.84–11.2)	<0.001
Lymphocyte (×10^3^/μL), median (IQR)	1.23 (0.64–1.71)	1.53 (1.11–2)	0.73 (0.44–1.39)	<0.001
Neutrophil (×10^3^/μL), median (IQR)	4.92 (3.01–8.37)	3.17 (2–4.92)	5.58 (3.96 -9.49)	<0.001
PLT (×10^3^/μL), median (IQR)	191 (147–254)	198 (174–248)	183 (98–232)	0.037
Total protein (g/L), median (IQR)	68.8 (63–73)	72 (68–74)	62 (53–67)	<0.001
Albumin (g/L), median (IQR)	37.8 (29.7–42.8)	43 (39–45)	32 (25–37)	<0.001
ProBNP (pg/mL), median (IQR)	134 (21–1102)	37.5 (13–137)	555 (90–1936)	<0.001
CRP (mg/L), median (IQR)	25.1 (4.5–111.9)	4.2 (2–10.5)	79.7 (35.6–150.3)	<0.001
Procalcitonin (ug/L), median (IQR)	0.061 (0.039–0.155)	0.052 (0.037–0.073)	0.233 (0.117–0.589)	<0.001
Ferritin (ug/L), median (IQR)	233 (68–505)	128 (42–254)	439 (186–1053)	<0.001
Creatinine (mg/dL), median (IQR)	0.94 (0.75–1.44)	0.78 (0.69–1)	0.95 (0.82–1.59)	<0.001
D-Dimer (μg/mL), median (IQR)	531 (295–1543)	343 (224–802)	924 (324–1570)	<0.001
Comorbidities				
Hypertension, *n* (%)	240 (46.1)	96 (31.1)	144 (67.9)	<0.001
Diabetes, *n* (%)	88 (16.9)	27 (8.7)	61 (28.8)	<0.001
Cigarette smoking, *n* (%)	187 (35.9)	98 (31.7)	89 (42)	0.016
Coronary artery disease, *n* (%)	54 (10.4)	16 (5.2)	38 (17.9)	<0.001
chronic heart failure, *n* (%)	24 (4.6)	4 (1.3)	20 (9.4)	<0.001
COPD, *n* (%)	151 (29)	63 (20.4)	88 (41.5)	<0.001
CKD (eGFR < 60 mL/min/m^2^), *n* (%)	66 (12.7)	22 (7.1)	44 (20.8)	<0.001
CVD, *n* (%)	39 (7.5)	7 (2.3)	32 (15.1)	<0.001

SBP, systolic blood pressure; DBP, diastolic blood pressure; RR, respiratory rate; Hgb, hemoglobin; WBC, white bloodcount; PLT, platelet; BNP, B type natriuretic peptide; CRP, C-reactive protein; COPD, chronic obstructive pulmonary disease; CKD, chronic kidney disease; CVD, cerebrovascular disease.

**Table 2:** Chest CT characteristics.

		Overall (*n* = 521)	Survivors (*n* = 309)	Non-Survivors (*n* = 212)	*p*-Value
**Typical, *n* (%)**		316 (60.7)	150 (48.5)	166 (78.3)	<0.001
	Bilateral, peripheral, and basal GGO with or without consolidation, *n* (%)	301 (57.8)	139 (45)	162 (76.4)	<0.001
	Bilateral, peripheral, and basalconsolidation, *n* (%)	58 (11.1)	27 (8.7)	31 (14.6)	0.036
	Multifocal rounded GGO with or without consolidation, *n* (%)	25 (4.8)	21 (6.8)	4 (1.9)	0.01
	Peribronchial enlargement, *n* (%)	42 (8.1)	20 (6.5)	22 (10.4)	0.108
	Reversed halo sign, *n* (%)	10 (1.9)	4 (1.3)	6 (2.8)	0.209
	Crazy paving pattern, *n* (%)	10 (1.9)	0 (0)	10 (4.7)	<0.001
**Indeterminate, *n* (%)**		61 (11.7)	43 (13.9)	18 (8.5)	0.058
	Unilateral GGO with or without consolidation, *n* (%)	40 (7.7)	34 (11)	6 (2.8)	0.001
	Perihilar GGO with or without consolidation, *n* (%)	4 (0.8)	0 (0)	4 (1.9)	0.015
	Diffuse GGO, *n* (%)	5 (1)	5 (1.6)	0 (0)	0.063
	Few and small non-peripheric GGO, *n* (%)	3 (0.6)	3 (1)	0 (0)	0.15
**Atypical, *n* (%)**		34 (6.5)	10 (3.2)	24 (11.3)	<0.001
	Lobar pneumonia, *n* (%)	4 (0.8)	2 (0.6)	2 (0.9)	0.704
	Effusion, *n* (%)	16 (3.1)	0 (0)	16 (7.5)	<0.001
	Interlobular septal thickening, *n* (%)	6 (1.2)	2 (0.6)	4 (1.9)	0.193
	Pulmonary fibrosis, *n* (%)	6 (1.2)	2 (0.6)	4 (1.9)	0.193
	Lymphadenopathy, *n* (%)	2 (0.4)	2 (0.6)	0 (0)	0.241
**Involved Lobes and global Score**					
	RUL, *n* (%)	297 (57)	132 (42.7)	165 (77.8)	<0.001
	RML, *n* (%)	242 (46.4)	98 (31.7)	144 (67.9)	<0.001
	RLL, *n* (%)	250 (47.9)	134 (43.3)	170 (80)	<0.001
	LUL, *n* (%)	305 (58.5)	129 (41.7)	176 (83)	<0.001
	LLL, *n* (%)	299 (57.4)	123 (39.8)	176 (83)	<0.001
	Global score, median (IQR)	5 (0–10)	1 (0–6)	10 (5–13)	<0.001

CT, computed tomography.

**Table 3:** Univariable and multivariable predictors of in-hospital mortality.

	Univariate Analysis		Multivariate Analysis	
	OR (95% CI)	*p*-Value	OR (95% CI)	*p*-Value
Age	1.12 (1.10–1.15)	<0.001	1.10 (1.02–1.18)	0.008
CRP	1.018 (1.015–1.02)	<0.001	1.013 (1.002–1.023)	0.015
Diabetes	4.21 (2.54–6.91)	<0.001	5.51 (1.77–17.2)	0.003
Global CT score	1.25 (1.19–1.30)	<0.001	1.73 (1.27–2.34)	<0.001

CRP, C-reactive protein; CT, computed tomography.

## Data Availability

Data supporting the reported results are available to the authors.
